# Interactions of *Bunias orientalis* plant chemotypes and fungal pathogens with different host specificity in vivo and in vitro

**DOI:** 10.1038/s41598-020-67600-7

**Published:** 2020-07-01

**Authors:** Lisa Johanna Tewes, Caroline Müller

**Affiliations:** Department of Chemical Ecology, Bielefeld University, Universitätsstr. 25, 33615 Bielefeld, Germany

**Keywords:** Ecology, Community ecology, Invasive species

## Abstract

Within several plant species, a high variation in the composition of particular defence metabolites can be found, forming distinct chemotypes. Such chemotypes show different effects on specialist and generalist plant enemies, whereby studies examining interactions with pathogens are underrepresented. We aimed to determine factors mediating the interaction of two chemotypes of *Bunias orientalis* (Brassicaceae) with two plant pathogenic fungal species of different host range, *Alternaria brassicae* (narrow host range = specialist) and *Botrytis cinerea* (broad host-range = generalist) using a combination of controlled bioassays*.* We found that the specialist, but not the generalist, was sensitive to differences between plant chemotypes in vivo and in vitro. The specialist fungus was more virulent (measured as leaf water loss) on one chemotype in vivo without differing in biomass produced during infection, while extracts from the same chemotype caused strong growth inhibition in that species in vitro. Furthermore, fractions of extracts from *B. orientalis* had divergent in vitro effects on the specialist versus the generalist, supporting presumed adaptations to certain compound classes. This study underlines the necessity to combine various experimental approaches to elucidate the complex interplay between plants and different pathogens.

## Introduction

Plants produce a multitude of defensive compounds that mediate interactions with attacking organisms from different taxa^1^. Generalists may be fended-off by (sets of) defence compounds effectively, while specialists instead use such compounds as host selection cues^2,3^. These distinct roles of individual compounds in interactions with different natural enemies, including herbivores and pathogens, is discussed to be one of the major drivers of the evolution of diverse plant defences^4–6^. A high variation in chemical defence profiles can be particularly found within plant species, in which so-called chemotypes are formed, that differ in the composition of a certain metabolite class^7–10^. Several studies address the responses of insect herbivores to distinct chemical defence profiles in plant families or species^8,11,12^. In contrast, only little is known about the role of phytochemical variation in interactions with pathogens (but see, e.g.^7^).

Various plant species of the Brassicaceae family show different chemotypes and are well-studied for interactions of certain chemotypes with plant enemies. For example, distinct chemotypes have been found in *Arabidopsis thaliana*^13,14^, *Barbarea vulgaris*^10,15^ and *Brassica oleracea*^16,17^. Interestingly, in *B. vulgaris* one chemotype is resistant to two herbivore species, while another is instead resistant to an oomycete pathogen^18^, underlining the importance to consider enemies from different taxa in chemo-ecological studies. Chemotypes in the Brassicaceae are typically characterised by variations in profiles of glucosinolates, a structurally highly diverse group of defence compounds^19^. Glucosinolates are assumed to be largely involved in resistance of Brassicales plants against generalist insect herbivores, whereas various specialists are adapted to these compounds^20^. Assuming that diversity in different compound classes may enhance the defence against various plant enemies, several other metabolites apart from glucosinolates as well as enzyme activities or mechanical traits may differ between chemotypes^21^. For example, in *B. oleracea*, glucosinolate profiles could not explain observed differences in susceptibility of distinct chemotypes to different herbivores^22^. Therefore, comprehensive, standardised approaches are essential to elucidate the numerous factors mediating interactions of plant chemotypes with natural enemies.

The Brassicaceae *Bunias orientalis* L. harbours two distinct genetic lineages, which represent two distinct chemotypes, that differ in their glucosinolate profiles and other polar metabolites^23^, as well as in susceptibility to plant enemies^23–25^. Plants from one chemotype, collected in the Caucasus region where the species potentially originates^26^, showed lower abundance of various herbivores and pathogens in a field common garden and were of lower host quality for a generalist herbivore in a laboratory experiment than plants of another chemotype, which was collected in the species’ invasive range in northern parts of Eurasia^23,25^. However, it is unclear whether differences in pathogen infection spot abundance ultimately represent the impact of infection on the plants and whether chemotype susceptibility of *B. orientalis* differs in dependence of the degree of host specificity of the pathogens.

Necrotrophic, asexual fungal plant pathogens are ubiquitous in air and soil. If fungal conidia successfully germinate and infect plant tissues, the fungal mycotoxins help to digest and consume these plant parts, ultimately leading to chlorosis, necrosis and wilting of leaves and/or plant death^27^. These necrotrophs can easily be cultivated in the laboratory and thus used for both in vivo and in vitro bioassays. *Alternaria brassicae* (Berk.) Sacc. (Pleosporaceae) occurs on a wide range of plant species almost exclusively within the Brassicaceae family (~ 640 records on ~ 90 species), including *B. orientalis*^28,29^. *Botrytis cinerea* Pers. (Sclerotiniaceae) has an extraordinary high host spectrum (> 3,000 records on > 200 species) including few members of the Brassicaceae (~ 90 records on ~ 20 species but no reports yet on *B. orientalis*^29,30^), and may thus moderately tolerate various classes of defence metabolites. These well-studied species could naturally come into contact with *B. orientalis*, and are thus suitable models to investigate interactions of chemotype traits with fungal attackers with potentially different adaptations to the host chemistry under controlled conditions. Due to their different host range, *A. brassicae* and *B. cinerea* are in the following referred to as ‘specialist’ and ‘generalist’, respectively.

In the present study, we used laboratory bioassays to characterise the performance of the fungal pathogen species *A. brassicae* and *B. cinerea* in response to plants from two *B. orientalis* populations from Turkey and Germany, belonging to the distinct chemotypes (‘Turkish’ versus ‘German’ chemotype). In an in vivo bioassay pre-damaged leaves were inoculated with pathogens and the degree of plant damage caused by fungal infection was estimated as leaf wilting by measuring leaf water loss. Fungal growth in vivo was measured by quantifying ergosterol as biomarker for fungal biomass in the leaves. To investigate the relevance of plant metabolites in the plant-fungus interaction, fungal growth was measured in vitro in culture broth amended with extracts from the two plant chemotypes using photometer assays. To characterise the activity of groups of metabolites influencing fungal growth, plant extracts were fractioned by polarity and fractions used for in vitro growth assays. We expected divergent effects of plant chemistry on the specialist versus the generalist fungal species. Overall, only a combination of bioassays may allow to pinpoint factors mediating *B. orientalis* interactions with pathogens.

## Results

### Visual infection differences between fungal species

**Figure 1:**
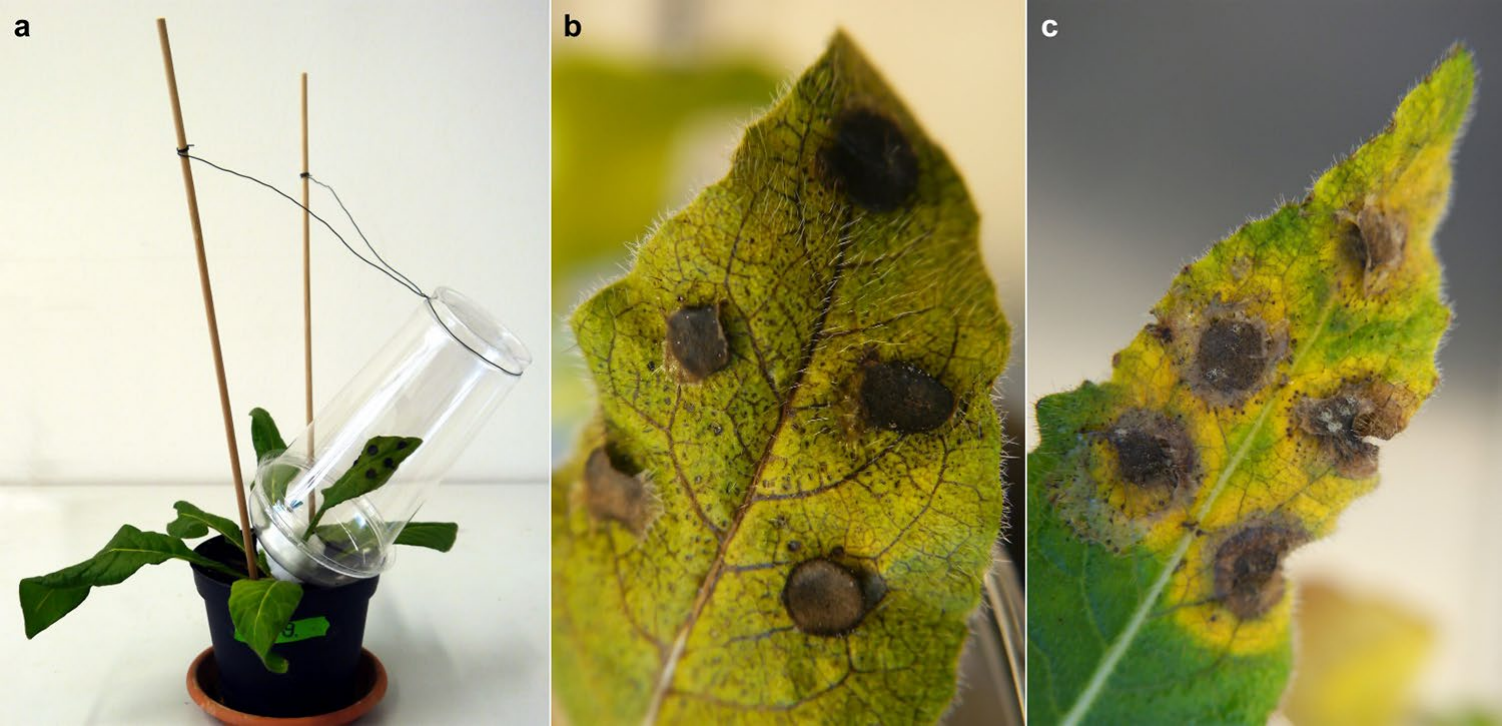
Photographs of in vivo bioassays with *Bunias orientalis* plants and the fungal pathogens *Alternaria brassicae* and *Botrytis cinerea*. (**a**) Experimental set-up: leaves were fixed in plastic cups with lids and five agar disks containing growing mycelium and conidia were applied with growth medium on mechanically damaged locations. Plastic cups were attached to wooden sticks with wire. (**b**, **c**) Typical patterns of severe infection on the upper side of the leaves five days after inoculation with (**b**) *A. brassicae* and (**c**) *B. cinerea* are shown. Photographs by Thorben Müller.

Plants of the two chemotypes from Turkey and Germany were inoculated with either *A. brassicae* or *B. cinerea* and incubated for eight days. Infection by both fungal species was observed in all *B. orientalis* plants from both chemotypes. However, *A. brassicae* and *B. cinerea* developed different infection patterns (Fig. 1b, c). Infection by *A. brassicae* was typically visible as necrotic leaf veins, which were wide-spread over the leaf blade (Fig. 1b), while in leaves infected by *B. cinerea* a circular necrotic spot developed directly around the inoculation area, which was surrounded by a chlorotic ring (Fig. 1c).

### Leaf water content and water loss upon infection

**Figure 2:**
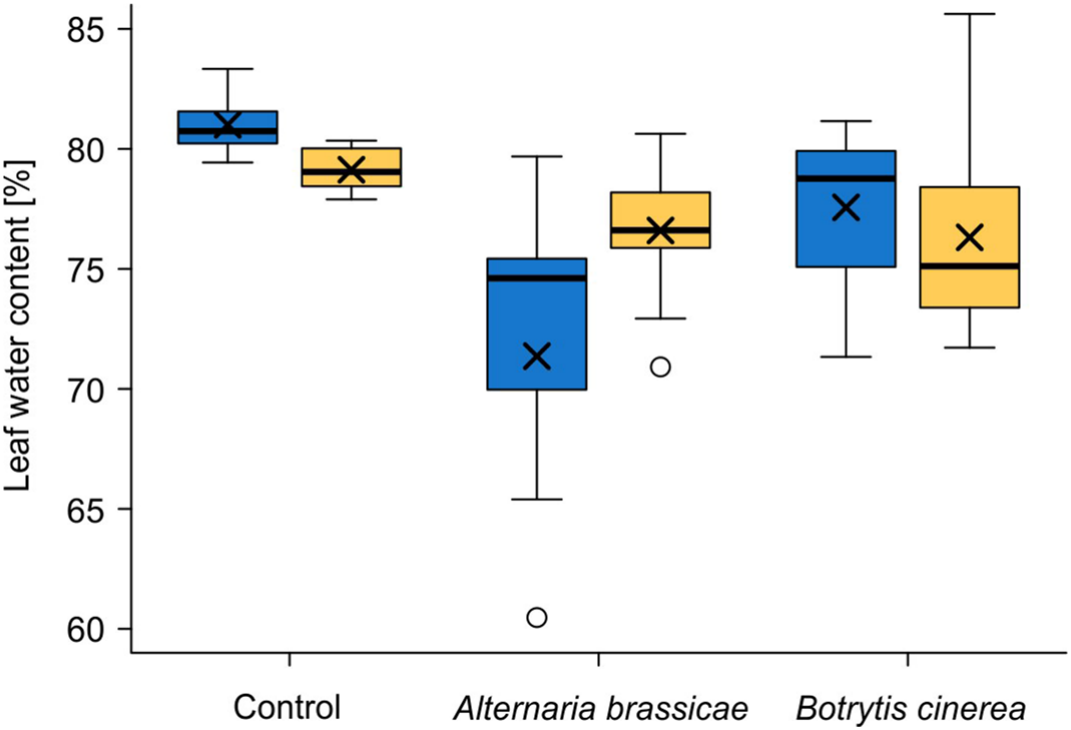
Water content in leaves of *Bunias orientalis* plants of different chemotypes, partly infected by fungal pathogens. Untreated control leaves from plants of Turkish (blue) and German chemotype (orange) were harvested before infection and leaves of five plants were pooled into one sample (n = 6 samples per chemotype). Water content in leaves infected by *Alternaria brassicae* or *Botrytis cinerea* was measured eight days after inoculation (n = 15 replicates per plant chemotype and fungal species). Boxes show medians, 25th and 75th percentiles, crosses show means, whiskers are minimum and maximum within 1.5-fold interquartile ranges, open dots are outliers. One outlier (46.9%) of the plants of the Turkish chemotype infected with *A. brassicae* is not shown.

Prior to inoculation, the water content measured in untreated control leaves was significantly higher in plants of the Turkish chemotype than in plants of the German chemotype (linear model, LM; df = 1, F = 7.826, P = 0.019, n = 6 replicates per plant chemotype; Fig. 2). After 8 days of incubation with the pathogens, the leaf water loss compared to the initial water content per chemotype was significantly affected by an interaction of the plant chemotype and the fungal species (linear mixed-effects model, LMM; df = 1, Chi^2^ = 6.296, P = 0.012, n = 15 per plant chemotype and fungal species). Infection of *A. brassicae* caused a higher water loss in plants of the Turkish chemotype than of the German chemotype, whereas water loss upon infection of *B. cinerea* did not differ between plants of both chemotypes (Fig. 2).

### Fungal biomass produced in vivo

The total amount of the fungal membrane component ergosterol was determined in each infected leaf to compare fungal biomass production of both species on plants of different chemotypes. Ergosterol could properly be separated and identified in all samples. A typical chromatogram of infected plant material and information on ergosterol recovery are given in Supplementary Information Fig. S1 and Methods S2, respectively. The recovery of ergosterol from fungal biomass differed between the fungal species as inferred from negative controls, being higher in *A. brassicae* than in *B. cinerea* (not shown)*.* Compared to negative controls and irrespective of plant chemotype an average increase of ergosterol amount of 40.5% for *A. brassicae* and of 8.7% for *B. cinerea* was detected in infected leaves. The fungal biomass present in/on infected leaves did not differ between plants of different chemotypes in both *A. brassicae* (LMM; df = 1, Chi^2^ = 1.016, P = 0.314, n = 15 per plant chemotype) and *B. cinerea* (LMM; df = 1, Chi^2^ = 0.138, P = 0.710, n = 15 per plant chemotype). Likewise there were no significant correlations between fungal virulence (i.e. leaf water loss) and fungal growth (i.e. ergosterol amount) for both *A. brassicae* (Spearman rank correlation S = 4,168; rho = 0.073; P = 0.702; n = 30) and *B. cinerea* (S = 3,606.5; rho = 0.198; P = 0.295; n = 30).

### Chemical composition of leaf extracts and fractions

**Figure 3:**
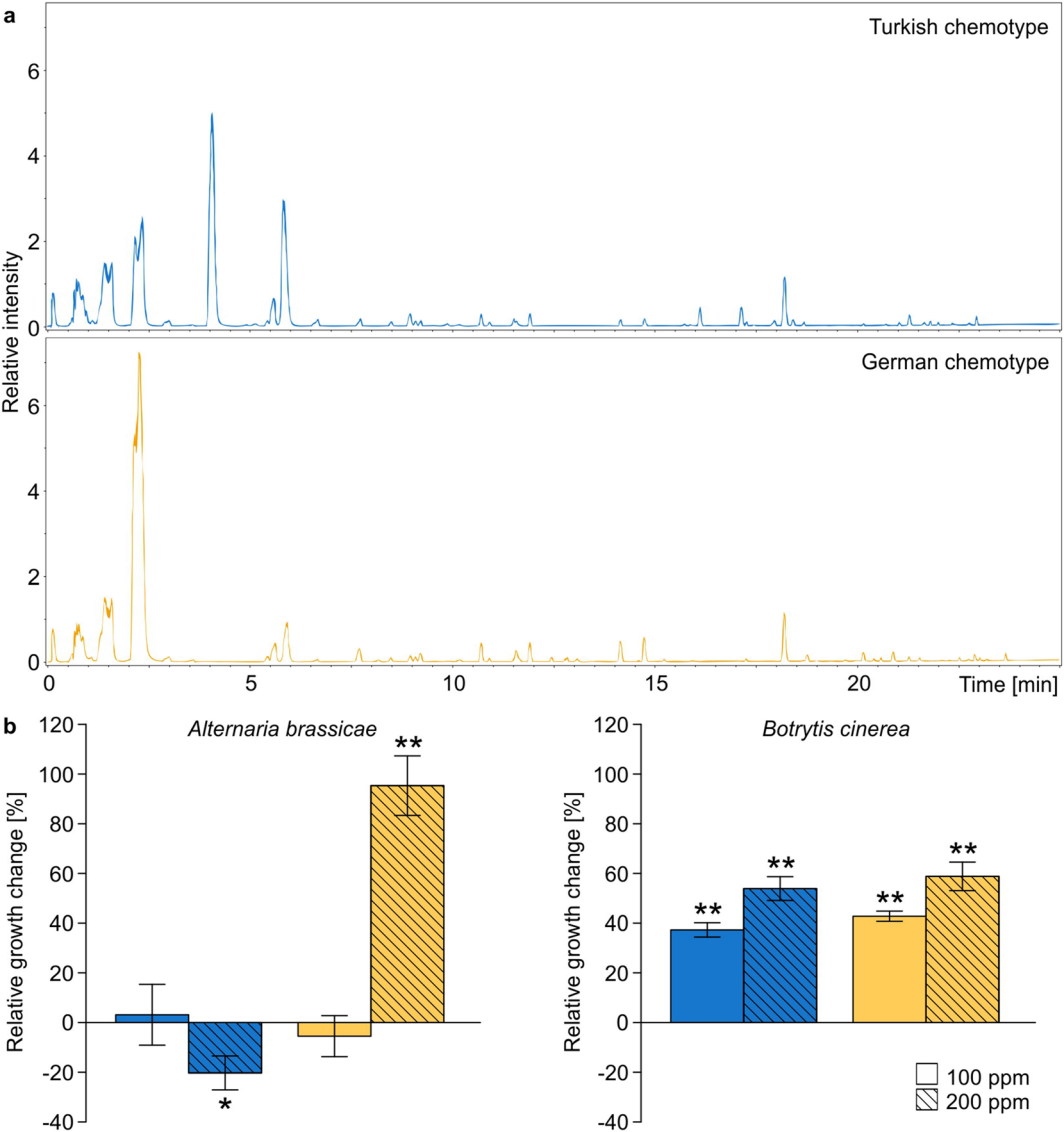
Chromatograms of leaf extracts from *Bunias orientalis* plants of different chemotypes and fungal growth response. (**a**) Composition of methanol leaf extracts from plants of Turkish (blue) and German chemotype (orange), analysed using ultra high performance liquid chromatography coupled with time-of-flight mass spectrometry. (**b**) Relative mycelium growth of the fungal pathogens *Alternaria brassicae* and *Botrytis cinerea* in liquid nutrient broth amended with the extracts in concentrations of 100 and 200 ppm. Fungal biomass was measured as optical density three days after subjecting conidia to amended nutrient broth and is presented as percentage of growth change in relation to means of control samples without plant extracts. Bars show means ± standard errors (n = 8–10 replicates). Asterisks indicate changes in growth being significantly (*P < 0.05, **P < 0.01) different from zero (i.e. the control mean values) in one-sided sign tests.

To characterise the composition of semi-polar metabolites of *B. orientalis* plants from different chemotypes, leaf extracts were prepared and analysed. Chemical analysis revealed clear differences in the overall metabolic composition of different *B. orientalis* chemotypes, inferred from combined leaf material of all plant individuals used per chemotype (n = 30 per chemotype) (Fig. 3a). Glucosinolate composition was determined from six leaf samples per chemotype with leaves pooled from five plants per sample. The composition of twelve glucosinolates, identified and quantified as in Tewes et al.^23^, largely differed between the two chemotypes, although the total glucosinolate concentration was comparable in both chemotypes (Table 1). Plants of the Turkish population were dominated by aliphatic glucosinolates, whereas in the German chemotype *p*-hydroxybenzyl glucosinolate was highly abundant (Table 1). The patterns correspond with findings from a previous study^23^ in which the metabolite and glucosinolate composition of those chemotypes were characterised on the plant individual level.

**Table 1:** Glucosinolate concentrations (mean ± SD) in leaves of *Bunias orientalis* plants of the Turkish and German chemotype with retention time (RT) in the chromatograms.

		Mean concentration (µmol g DW^−1^)	
Glucosinolate (GS)	RT [min]	Turkish	German
*n*-Butyl GS^A^	3.9	23.29 ± 4.29	0.005 ± 0.004^(4)^
1-Methylethyl GS^A^	2.2	12.77 ± 2.49	0.001 ± 0.002^(2)^
4-Methylsulfinyl-3-butyl GS^A^	1.5	11.43 ± 2.24	9.94 ± 2.02
4-Methylsulfinylbutyl GS^A^	1.4	3.66 ± 2.11	4.84 ± 0.90
4-Methylthio-3-butenyl GS^A^	5.7	5.13 ± 1.30	1.47 ± 0.48
4-Methylthiobutyl GS^A^	5.4	0.897 ± 0.259	0.668 ± 0.232
5-Methylthiopentyl GS^A^	8.0	0.012 ± 0.007^(5)^	0.001 ± 0.002^(1)^
3-Butenyl GS^A^	2.8	0.002 ± 0.006^(1)^	n.d.
*p*-Hydroxybenzyl GS^B^	2.2	21.33 ± 5.77	64.37 ± 4.12
Benzyl GS^B^	5.2	0.002 ± 0.004^(2)^	0.007 ± 0.004
4-Methoxyindol-3-ylmethyl GS^I^	8.7	0.771 ± 0.164	0.357 ± 0.041
Indol-3-ylmethyl GS^I^	6.2	0.013 ± 0.011	0.010 ± 0.007^(5)^
Total		79.22 ± 10.79	81.66 ± 5.65

From 30 plants per chemotype, one leaf per plant was harvested and five leaves were pooled into one sample (n = 6 samples per chemotype). Superscript upper case letters denote the side chain types of the glucosinolates as aliphatic (‘A’), benzenic (‘B’) and indole (‘I’). Superscript numbers in brackets denote the number of samples out of six per chemotype in which the glucosinolate was detected, if not found in all samples per chemotype. Not detected in any sample per chemotype is denoted as ‘n.d.’

**Figure 4:**
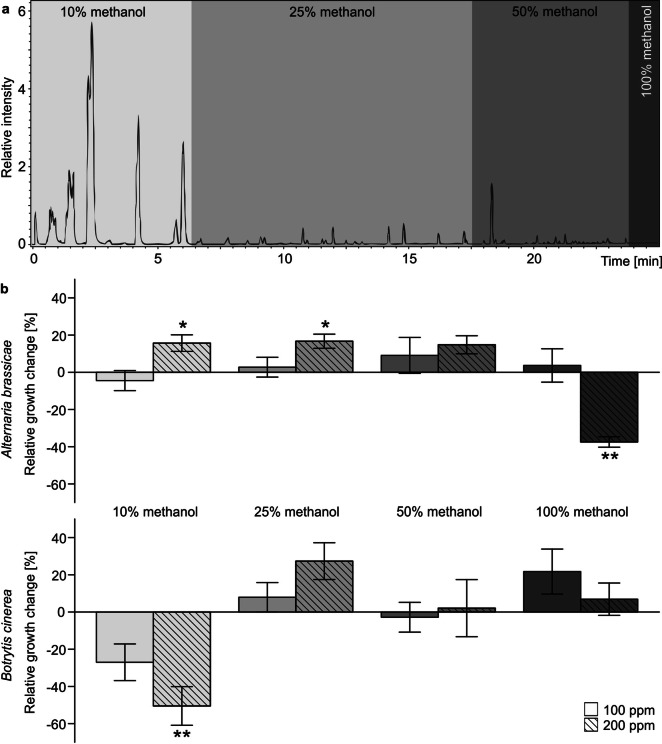
Chromatogram of a leaf extract from *Bunias orientalis* plants and fungal growth response. (**a**) Composition of methanol extracts from leaves of plants mixed from different chemotypes, analysed using ultra high performance liquid chromatography coupled with time-of-flight mass spectrometry. Fractions of the leaf extract that were aimed to be separated by solid phase extraction are marked in different greyscales. See Supplementary Information Fig. S2 for details on the fractionation results. (**b**) Relative mycelium growth of the fungal pathogens *Alternaria brassicae* and *Botrytis cinerea* in liquid nutrient broth amended with the fractions of leaf extract in concentrations of 100 and 200 ppm. Fungal biomass was measured as optical density three days after subjecting conidia to amended nutrient broth and is presented as percentage of growth change in relation to means of control samples without plant extracts. Bars show means ± standard errors (n = 7–10 replicates). Asterisks indicate changes in growth being significantly (*P < 0.05, **P < 0.01) different from zero (i.e. the control mean values) in one-sided sign tests.

To separate classes of potentially active compounds present in *B. orientalis* leaves, methanol leaf extracts, in which plant material from both chemotypes was combined, were used. Extracts were fractionated using solid phase extraction with four different methanol:water mixtures as eluents and chemically analysed. The four fractions of the extract differed in their dry mass as well as in their presumed (Fig. 4a) and measured (Supplementary Information Fig. S2) chemical composition. Glucosinolates were mainly present in the 10% methanol fraction (Table 1, Fig. S2a) which represented ~ 11.7% of the extracted leaf dry mass. The 25% methanol fraction (Fig. S2b) represented ~ 1.6% of the leaf dry mass and contained few glucosinolates and putative flavonoids, based on their UV spectra recorded at 360 nm (not shown). The 50% methanol fraction (~ 2.6% of leaf dry mass) partly overlapped with both the 25% and the 100% methanol fractions (Fig. S2b–d) and contained putatively flavonoids and yet unidentified metabolites. The 100% methanol fraction (~ 5.5% of leaf dry mass) contained mainly metabolites that could not be detected using the underlying analytical method (Fig. S2d).

### Fungal growth in vitro

To estimate the effects of semi-polar metabolites of *B. orientalis* plants from different chemotypes on fungal growth of both species, leaf extracts were prepared and used for amendment of liquid culture broth in which fungal growth was measured photometrically. Testing the effects of extracts from leaf material pooled from all plants within chemotypes on fungal growth, biomass produced by *A. brassicae* was significantly affected by plant chemotype in high extract concentration (LM; df = 1, F = 70.251, P < 0.001, n = 9 per plant chemotype). Compared to the control, a significant growth inhibition was caused by extracts from the Turkish chemotype, while a growth facilitation was found in response to extracts of the German chemotype plants (Fig. 3b; for detailed statistical results on fungal growth change in response to all extracts and extract fractions see Supplementary Information Table S1). In contrast, in low extract concentration no fungal biomass differences in response to plant chemotypes were found (LM; df = 1, F = 0.306, P = 0.588, n = 8–10 per plant chemotype), and likewise extracts caused no significant changes in growth compared to the control (Fig. 3b). Biomass produced by *B. cinerea* did not differ in presence of extracts from plants of different chemotypes in both high (LM; df = 1, F = 0.433, P = 0.519, n = 10 per plant chemotype) and low (LM; df = 1, F = 2.520, P = 0.131, n = 9–10 per plant chemotype) extract concentrations. Compared to the control, all extracts caused significant growth facilitation in *B. cinerea*, irrespective of plant chemotype and extract concentration (Fig. 3b). However, on average, growth facilitation seemed overall higher in high extract concentration, and slightly lower in extracts from plants of the Turkish than of the German chemotype (Fig. 3b).

To localise classes of *B. orientalis* metabolites that influence the specialist versus the generalist fungus in particular, four fractions from solid phase extraction of extracts from leaf material from both chemotypes was combined, were used for amendment of liquid culture broth for fungal growth assays. The glucosinolate-containing 10% methanol fraction caused significant growth facilitation in *A. brassicae* when tested in high concentration, but significant growth inhibition in *B. cinerea* compared to the control (Fig. 4b). The 25% methanol fraction in high concentration caused on average growth facilitation in both fungal species, which was significant for *A. brassicae* (Fig. 4b). The 50% methanol fraction caused no significant changes in growth of both fungal species (Fig. 4b). The 100% methanol fraction in high concentration caused significant growth inhibition in *A. brassicae* but not in *B. cinerea* (Fig. 4b).

## Discussion

In the present study, pre-damaged leaves of both *B. orientalis* chemotypes were susceptible to both *A. brassicae* and *B. cinerea,* indicated by chlorosis, necrosis and water loss of leaves*.* However, the degree of plant damage, i.e. leaf water loss, was affected by an interaction of plant chemotype and fungal species. When plants were infected with the specialist, plants from the Turkish chemotype had a higher water loss and thus likely suffered more from infection. This suggests that virulence of *A. brassicae*, but not of *B. cinerea*, is highly sensitive to intraspecific variation in the host metabolite profile. In addition to chemical traits, morphological leaf traits may influence fungal development and can differ between plant chemotypes, as in this study found for the leaf water content. Thus, various characteristics of both different *B. orientalis* chemotypes and fungal species with different host specificity may determine the outcome of this in vivo interaction.

Various constitutive plant (defence) metabolites extractable in methanol and tested for their effect on fungal growth in vitro may be involved in divergent chemotype interactions with the specialist fungus, as discussed below. In addition, phytoalexins may have been induced differently in both chemotypes. These rapidly induced metabolites play an important role in anti-pathogen defence^31–33^, including defence against both *A. brassicae*^34,35^ and *B. cinerea*^36,37^.

Importantly, not only plants but also pathogens produce a wide range of metabolites, of which some may be responsible for the differences in plant damage caused by the two fungus species on plants of different *B. orientalis* chemotypes. Mycotoxins damage host plant cells, which is required for fungi to thrive on the plant tissue^27^. In *A. brassicae* the cyclodepsipeptide ‘destruxin B’ is a main factor determining disease development^38,39^. Effects of destruxin B have been found to be host-selective^40^, e.g. due to differing efficiency of enzymatic detoxification by the host plant^41^, and may also differ in the impact on distinct chemotypes within *B. orientalis*. In contrast, in *B. cinerea* the non-specific sesquiterpene ‘botrydial’ acts as virulence factor in interaction with numerous host plant species^30,42,43^. The lack of specificity in this mycotoxin fits with the lack of virulence differences between plant chemotypes in the present study.

Interestingly, the patterns found in plant damage were not mirrored in the fungal biomass produced after infection in vivo, which did not differ between plant chemotypes, irrespective of fungal species. Nevertheless, the high recovery of ergosterol from leaf material infected by *A. brassicae* and *B. cinerea* suggests ergosterol as promising biomarker for biomass of these and other eufungal pathogens in infected leaf material. Comparing fungal growth in vitro in presence of raw leaf extract of plants from the two *B. orientalis* chemotypes, a divergent influence of plant chemotype on different fungal species was revealed, as only the specialist growth was affected. This finding is in line with the high sensitivity towards chemotype differences suggested above for *A. brassicae* in in vivo assays. Compared to the control *A. brassicae* showed significant growth inhibition versus growth facilitation by metabolites present in leaf extracts from the Turkish versus the German chemotype. Regarding the invasion history of *B. orientalis*, such differences in chemical defence may have been shaped by random genetic drift after population founding^23^ or by distinct selection pressures the plants experienced in their native (Turkish) versus the introduced (German) range^25^. A reduced pressure by different specialist enemies in novel habitats may have led to changes in the chemical defence composition in plants of the German population^44,45^. However, growth of the broad generalist *B. cinerea* was not sensitive towards such chemotypic differences, underlining that controlled bioassays should ideally be conducted with several natural enemy species of distinct host specificity.

In the fractions of leaf extract pooled from both *B. orientalis* chemotypes differences in susceptibility between fungal species were found, which can be linked with the fungal host specificity. Most strikingly, the glucosinolate containing 10% methanol fraction caused growth facilitation in the Brassicaceae specialist but growth inhibition in the generalist. Indeed, a rather high tolerance of glucosinolate breakdown products in vitro, and also a positive correlation of infection and glucosinolate content in vivo has been revealed in *Alternaria* species specialised to Brassicaceae^2,35,47^. The generalist *B. cinerea* has been found to be sensitive to these compounds^37,46^, although tolerance of some glucosinolate breakdown products has been suggested^47^. Potential flavonoids and/or other metabolites present in the 25% and 50% methanol fractions did not lead to remarkably divergent growth responses in both fungi. In contrast, one or several compounds only soluble in 100% methanol were likely relevant for *B. orientalis* defence against *A. brassicae* but not against *B. cinerea*. Further bioassay-guided fractionation and analytical techniques are needed to isolate and identify these compounds.

The patterns observed in this study suggesting differing susceptibility of the two *B. orientalis* chemotypes to the two fungal species were not consistent across experimental approaches. The increased virulence of *A. brassicae* when infecting the Turkish chemotype in vivo contradicts the higher defence potential of metabolites from that chemotype against this specialist fungus in vitro*.* Similarly, *A. thaliana* mutants with a novel glucosinolate were more susceptible to *Alternaria brassicicola* infection than wild-type plants in vivo, although breakdown products of that glucosinolate were growth inhibitory in vitro^48^, underlining that plant-fungus interactions are determined by an interplay of various factors, rather than by the direct toxicity of certain metabolites. Furthermore, the differences in results on fungal growth in vivo and in vitro in the present study may be linked to the late (8 days) and early (3 days) time points of growth measurement, respectively. While plant chemotype differences seemed to play a role for conidia germination and/or growth in early stages of the infection process, produced biomass may be more balanced after a certain time period of successful infection of the plant.

A previous study suggested lower susceptibility of plants from the Turkish than of the German chemotype to unidentified pathogens when grown under field common garden conditions^25^. Contrasting findings in the present in vivo experiments may be partly related to the influence of mechanical leaf barriers that were to some extent reduced by pre-damaging the leaves prior to infection. As mechanical barriers, epicuticular wax layers can largely determine plant resistance against fungal pathogens^49,50^, including *A. brassicae*^51^ and *B. cinerea*^36^*.* Moreover, the density and shape of trichomes can be crucial for plant resistance against various plant attackers^36,52,53^. Indeed, trichome patterns highly differ between *B. orientalis* populations from the Turkish versus the German chemotype^24^. This suggests an important role of mechanical leaf defence traits for susceptibility of *B. orientalis* chemotypes in natural scenarios.

Taken together, the examined interactions of *B. orientalis* chemotypes and two fungal pathogen species revealed to be highly complex, potentially depending on the leaf structure and the specific impacts of various plant and fungal metabolites. We could highlight the pronounced influence of fungal host specificity on the fungal response to plant chemotype differences. Overall, our study underlines that the outcomes of different types of field studies and laboratory bioassays strongly depend on the various factors in- or excluded within the experiment. The combination of different types of bioassays helps to avoid misinterpretations and to gain insights in factors influencing individual plant-fungus interactions.

## Methods

### Plant and pathogen rearing

Plants originated from a population in Turkey (T4; near Rize, 40° 44.33′ N, 40° 44.12′ E) and another in Germany (JE; Jena, 50° 52.42′ N, 11° 44.76′ E), here termed ‘Turkish’ and ‘German’ chemotype. Populations were chosen as representatives of their chemotype groups and as they exhibited the highest differences in infection by pathogens in previous field observations^25^. Per chemotype, a total of 30 seedlings were germinated and used for the experiments. For details on plant rearing see Supplementary Information Methods S1.

The fungal pathogens *A. brassicae* (strain CBS 102.24) and *B. cinerea* (strain CBS 116760) were obtained from Westerdijk Fungal Biodiversity Institute (Utrecht, The Netherlands). Prior to the experiments, both species were cultivated in Petri dishes on a 5 mm layer of low strength potato dextrose agar growth medium (30 g L^−1^, Carl Roth, Karlsruhe, Germany) with 10 µg L^−1^ ZnSO_4_ × 7H_2_O and 5 µg L^−1^ CuSO_4_ × 5H_2_O. Conidia production was induced in a climate cabinet with a 1:1 mixture of white light and black light (8:16 h light:dark cycle) at 20 °C.

### In vivo assay of plant damage and fungal biomass production

Five days prior to inoculation, one leaf from the youngest fully developed leaf pair was chosen for later inoculation and the other leaf from the pair was harvested, weighed, frozen in liquid nitrogen and stored at − 80 °C. Before weighing, leaves of five plants were pooled into one sample (material of n = 30 plants in n = 6 samples per chemotype). These samples were lyophilised to calculate the water content in untreated leaves and to prepare samples of leaf material for chemical analysis and in vitro bioassays (see below). For each fungal species 30 plants (n = 15 per chemotype) were inoculated between 1 and 5 pm. Per fungal species, five culture plates were used and per plate 42 discs (6.7 mm diameter) with growing mycelium with conidia were taken. Additionally, six replicates of five pooled discs per fungal species were weighed and frozen at − 80 °C as negative control samples for determination of ergosterol content.

Prior to inoculation the target leaf was slid in the 25 mm hole of the lid from a plastic cup and the opening sealed with cotton wool (Fig. 1a). The leaves were mechanically damaged five times using a tool of four combined needles (0.4 mm diameter, arranged in a square of 3 × 3 mm). Subsequently, 20 µL of a growth medium, containing 26.5 g L^−1^ potato dextrose broth (Carl Roth) and 0.03% [w/v] Triton X-100 (Sigma-Aldrich, Steinheim, Germany) in millipore water, was spread on the damaged area and five discs from the fungal cultures were gently pressed upside down on that area. After inoculation, plastic cups were placed on the lids (total volume: 800 mL) and fixed to a wooden stick with wire (Fig. 1a). Eight days after inoculation, the tips of the infected leaves were cut below areas in which fungal infection was visible. All samples were weighed, frozen in liquid nitrogen and stored at − 80 °C. After lyophilisation, samples were weighed again to calculate the water content. The water loss of infected leaves was calculated by relating the water content of each leaf to the water content of the respective pooled untreated control leaves of the same plant population (i.e. one control per five samples).

Ergosterol is a mycosterine specific for fungal cell membranes and not present in plants, and thus a suitable biomarker for fungal biomass^54,55^. For ergosterol extraction, the lyophilised infected leaf samples and negative controls were pulverised and samples extracted threefold in methanol. The combined supernatants were concentrated and analysed using high performance liquid chromatography coupled with a diode array detector (1260 and 1290 Series, Agilent, Santa Clara, CA, USA). The UV-absorption of ergosterol was quantified at 282 nm and related to a commercial ergosterol reference standard solved in 100% methanol. For details on the extraction and instrument settings see Supplementary Information Methods S2.

### In vitro assay of fungal growth

Photometric measurement of mycelium produced in amended culture broth is a robust, small-scale in vitro method to investigate the effects of chemicals on fungal growth^56^. For bioassays, leaf material harvested from the experimental plants prior to infection was used and pooled for each of the two chemotypes. Six technical replicates per chemotype of 30 mg leaf material were extracted three times in 0.6 mL methanol, dried at 35 °C and the dry mass of the extract was determined. For extract fractionation dried extracts from leaf material, combined from both chemotypes, were suspended in 10% methanol in millipore water and applied to solid phase extraction columns (Chromabond C8, 1 mL reservoir, 100 mg bed weight, Macherey-Nagel, Düren, Germany). Columns were eluted with different methanol:water mixtures of decreasing polarity, resulting in 10%, 25%, 50% and 100% methanol fractions of the leaf extract, which were dried and weighed. For all extracts and extract fractions blank samples were prepared, which were treated like the test samples, but contained no leaf material. To determine the chemical composition of extracts and extract fractions, further samples were analysed using ultra high performance liquid chromatography coupled to a diode array detector (Dionex UltiMate 3000, Thermo Fisher, San José, CA, USA) and a quadrupole time of flight mass spectrometer (compact, Bruker Daltonics, Bremen, Germany). Therefore, extracts were prepared from healthy leaf material harvested to calculate the water content as described above (material of n = 30 plants pooled in n = 6 samples per chemotype). In addition, individual extracts of leaf material in the same composition as used in the bioassays, i.e., leaf material pooled from all plants per chemotype, and fractions of extracts from leaf material pooled from all plants including both chemotypes, were prepared. Samples were extracted in 90% methanol and chemical analysis was performed according to Schrieber et al. (2019)^57^, but in negative electrospray ionisation mode and with further modifications of instrument settings and in processing raw data from chromatograms (see Supplementary Information Methods S3). UV absorption was recorded at 360 nm. Glucosinolates were identified using their mass spectra and quantified according to Tewes et al. (2018)^23^ (for details see Supplementary Information Methods S3).

Plant extracts and extract fractions were suspended in 100% methanol in a concentration of 10,000 ppm, based on the extract or fraction dry mass. These extracts, extract fractions or blanks were added to potato dextrose broth in a 1:10 ratio and the solvent was evaporated at 50 °C for 1 h. The amended culture broth was further diluted to 100 ppm or 200 ppm plant extract concentration. For each extract or extract fraction in each concentration, ten bioassay replicates were set-up in 96-well photometer plates, whereby 180 µL of amended culture broth was applied to each test well. Control samples without plant metabolites were prepared likewise from blank samples with 10–20 replicates per extract or fraction and concentration.

Fungal conidia used for bioassays were harvested from culture plates in potato dextrose broth with 0.03% Triton X-100. Depending on conidia size, the conidia suspensions were filtered through two (*A. brassicae*) or three (*B. cinerea*) layers of Miracloth tissue (Millipore Corp., Billerica, MA, USA) and diluted to a concentration of approximately 0.5 × 10^5^ conidia per mL (*A. brassicae*) or 5 × 10^5^ conidia per mL (*B. cinerea*). To each test well with amended culture broth 20 µL of conidia suspension were added and the plates were sealed and incubated in the dark while shaking. Optical density of test wells was measured on a multiplate reader at 492 nm at 0 and 72 h after preparation. To calculate relative fungal growth for each replicate the background value at 0 h was subtracted from the 72 h value. The individual values were further related to the mean value of the control replicates to calculate the percentage of relative change in growth. Samples in which aerial mycelium was produced were not suitable for photometrical measurements and thus excluded from the analysis.

### Statistical analysis

All statistical analyses and figures were done with R (version 3.5.2^58^). To compare the water content of untreated control leaves between plants of different chemotypes, a LM (i.e. one-factorial ANOVA with *F* test) was performed. To analyse the influence of plant chemotype and fungal species on the water loss of infected leaves, a LMM was calculated (*lme4* package^59^), using culture plate identity (1–5) nested within fungal species as random factor. The total ergosterol amount in infected leaves was compared between plant chemotypes within each fungal species with LMMs, using the culture plate identity and the day of the extraction procedure (1–3) as random factors. In all LMMs response variables were transformed prior to the analysis, whereby arcus sinus square-root transformation was used for water loss, and log-transformation was used for ergosterol amount. LMMs were fitted with a maximum likelihood approach and P-values revealed based on likelihood ratio tests (Chi^2^ tests). Relationships between fungal damage and fungal growth were evaluated by calculating Spearman rank correlations for the untransformed raw data of water loss and ergosterol amount for each fungal species separately.

To investigate whether extracts from plants of different chemotypes differently influence fungal growth in vitro, separate LMs were calculated for different extract concentrations (100 ppm, 200 ppm) within each fungal species. To analyse whether amendment of culture broth with different plant extracts or extract fractions influences fungal growth relative to the controls, the relative inhibition values of replicates within treatments were investigated for being significantly different from zero (i.e. the control mean values) using one-sided sign tests (*BSDA* package^60^). The residuals of LMs and LMMs were inspected visually and analysed for normality and homoscedasticity with the Shapiro–Wilk test and the Levene test (*car* package^61^), respectively, and did not reveal obvious deviations from these assumptions.

## Supplementary information

Supplementary information

## Data Availability

Data associated with this paper will be accessible via https://pub.uni-bielefeld.de.

## References

[1] Bennett RN, Wallsgrove RM (1994). Secondary metabolites in plant defense-mechanisms. New Phytol..

[2] Giamoustaris A, Mithen R (1997). Glucosinolates and disease resistance in oilseed rape (*Brassica napus* ssp. *oleifera*). Plant Pathol..

[3] Schoonhoven LM, Van Loon JJA, Dicke M (2005). Insect-plant biology.

[4] van der Meijden E (1996). Plant defence, an evolutionary dilemma: Contrasting effects of (specialist and generalist) herbivores and natural enemies. Entomol. Exp. Appl..

[5] Maor R, Shirasu K (2005). The arms race continues: Battle strategies between plants and fungal pathogens. Curr. Opin. Microbiol..

[6] Bednarek P, Osbourn A (2009). Plant-microbe interactions: Chemical diversity in plant defense. Science.

[7] Macel M, Klinkhamer PGL (2010). Chemotype of *Senecio jacobaea* affects damage by pathogens and insect herbivores in the field. Evol. Ecol..

[8] Kleine S, Müller C (2011). Intraspecific plant chemical diversity and its effects on herbivores. Oecologia.

[9] Züst T (2012). Natural enemies drive geographic variation in plant defenses. Science.

[10] Christensen S (2014). Different geographical distributions of two chemotypes of *Barbarea vulgaris* that differ in resistance to insects and a pathogen. J. Chem. Ecol..

[11] Poelman EH, van Dam NM, van Loon JJA, Vet LEM, Dicke M (2009). Chemical diversity in *Brassica oleracea* affects biodiversity of insect herbivores. Ecology.

[12] Richards LA (2015). Phytochemical diversity drives plant–insect community diversity. Proc. Natl. Acad. Sci. USA.

[13] Arany AM (2008). Glucosinolates and other metabolites in the leaves of *Arabidopsis thaliana* from natural populations and their effects on a generalist and a specialist herbivore. Chemoecology.

[14] Bidart-Bouzat MG, Kliebenstein DJ (2008). Differential levels of insect herbivory in the field associated with genotypic variation in glucosinolates in *Arabidopsis thaliana*. J. Chem. Ecol..

[15] van Leur H, Vet LEM, Van der Putten WH, van Dam NM (2008). *Barbarea vulgaris* glucosinolate phenotypes differentially affect performance and preference of two different species of lepidopteran herbivores. J. Chem. Ecol..

[16] Gols R (2008). Genetic variation in defense chemistry in wild cabbages affects herbivores and their endoparasitoids. Ecology.

[17] Harvey JA, van Dam NM, Raaijmakers CE, Bullock JM, Gols R (2011). Tri-trophic effects of inter- and intra-population variation in defence chemistry of wild cabbage (*Brassica oleracea*). Oecologia.

[18] van Mölken T (2014). Consequences of combined herbivore feeding and pathogen infection for fitness of *Barbarea vulgaris* plants. Oecologia.

[19] Agerbirk N, Olsen CE (2012). Glucosinolate structures in evolution. Phytochemistry.

[20] Hopkins RJ, van Dam NM, van Loon JJA (2009). Role of glucosinolates in insect–plant relationships and multitrophic interactions. Annu. Rev. Entomol..

[21] Renwick JAA (2002). The chemical world of crucivores: lures, treats and traps. Entomol. Exp. Appl..

[22] Poelman EH, Galiart R, Raaijmakers CE, van Loon JJA, van Dam NM (2008). Performance of specialist and generalist herbivores feeding on cabbage cultivars is not explained by glucosinolate profiles. Entomol. Exp. Appl..

[23] Tewes LJ, Michling F, Koch MA, Müller C (2018). Intracontinental plant invader shows matching genetic and chemical profiles and might benefit from high defence variation within populations. J. Ecol..

[24] Fortuna TM (2014). Variation in plant defences among populations of a range-expanding plant: Consequences for trophic interactions. New Phytol..

[25] Tewes LJ, Müller C (2018). Syndromes in suites of correlated traits suggest multiple mechanisms facilitating invasion in a plant range-expander. NeoBiota.

[26] Koch MA (2017). Early-Mid Pleistocene genetic differentiation and range expansions as exemplified by invasive Eurasian *Bunias orientalis* (Brassicaceae) indicates the Caucasus as key region. Sci. Rep..

[27] Möbius N, Hertweck C (2009). Fungal phytotoxins as mediators of virulence. Curr. Opin. Plant Biol..

[28] Thomma BPHJ (2003). *Alternaria* spp.: From general saprophyte to specific parasite. Mol. Plant Pathol..

[29] Farr, D. F. & Rossman, A. Y. Fungal Databases, U.S. National Fungus Collections, ARS, USDA. Accessed 8 June 2019 https://nt.ars-grin.gov/fungaldatabases/ (2019).

[30] Williamson B, Tudzynsk B, Tudzynski P, van Kan JAL (2007). *Botrytis cinerea*: the cause of grey mould disease. Mol. Plant Pathol..

[31] Ahuja I, Kissen R, Bones AM (2012). Phytoalexins in defense against pathogens. Trends Plant Sci..

[32] Ribera AE, Zuñiga G (2012). Induced plant secondary metabolites for phytopatogenic fungi control: A review. J. Soil Sci. Plant Nutr..

[33] Pedras MSC, Yaya EE, Glawischnig E (2011). The phytoalexins from cultivated and wild crucifers: Chemistry and biology. Nat. Prod. Rep..

[34] Conn KL, Tewari JP, Dahiya JS (1988). Resistance to *Alternaria brassicae* and phytoalexin-elicitation in rapeseed and other crucifers. Plant Sci..

[35] Sellam A, Iacomi-Vasilescu B, Hudhomme P, Simoneau P (2007). In vitro antifungal activity of brassinin, camalexin and two isothiocyanates against the crucifer pathogens *Alternaria brassicicola* and *Alternaria brassicae*. Plant Pathol..

[36] Elad Y, Evensen K (1995). Physiological aspects of resistance to *Botrytis cinerea*. Phytopathology.

[37] Kliebenstein DJ, Rowe HC, Denby KJ (2005). Secondary metabolites influence Arabidopsis/*Botrytis* interactions: Variation in host production and pathogen sensitivity. Plant J..

[38] Ayer WA, Pena-Rodriguez LM (1987). Metabolites produced by Alternaria brassicae, the black spot pathogen of canola. Part 1, the phytotoxic components. J. Nat. Prod..

[39] Bains PS, Tewari JP (1987). Purification, chemical characterization and host-specificity of the toxin produced by *Alternaria brassicae*. Physiol. Mol. Plant Pathol..

[40] Buchwaldt L, Green H (1992). Phytotoxicity of destruxin B and its possible role in the pathogenesis of *Alternaria brassicae*. Plant Pathol..

[41] Pedras MSC, Khallaf I (2012). Molecular interactions of the phytotoxins destruxin B and sirodesmin PL with crucifers and cereals: Metabolism and elicitation of plant defenses. Phytochemistry.

[42] van Kan JAL (2006). Licensed to kill: The lifestyle of a necrotrophic plant pathogen. Trends Plant Sci..

[43] Choquer M (2007). *Botrytis cinerea* virulence factors: New insights into a necrotrophic and polyphageous pathogen. FEMS Microbiol. Lett..

[44] Doorduin LJ, Vrieling K (2011). A review of the phytochemical support for the shifting defence hypothesis. Phytochem. Rev..

[45] Müller C, Jeschke JM, Heger T (2018). Evolution of competitive ability and shifting defence hypotheses. Invasion Biology: Hypotheses and Evidence.

[46] Ugolini L, Martini C, Lazzeri L, D'Avino L, Mari M (2014). Control of postharvest grey mould (*Botrytis cinerea* Per.: Fr.) on strawberries by glucosinolate-derived allyl-isothiocyanate treatments. Postharvest Biol. Technol..

[47] Tierens KFMJ (2001). Study of the role of antimicrobial glucosinolate-derived isothiocyanates in resistance of *Arabidopsis* to microbial pathogens. Plant Physiol..

[48] Brader G, Mikkelsen MD, Halkier BA, Palva ET (2006). Altering glucosinolate profiles modulates disease resistance in plants. Plant J..

[49] Juniper BE, Andrews JH, Hirano SS (1991). The leaf from the inside and the outside: a microbe's perspective. Microbial Ecology of Leaves.

[50] Müller C, Riederer M (2005). Review: Plant surface properties in chemical ecology. J. Chem. Ecol..

[51] Meena PD, Awasthi RP, Chattopadhyay C, Kolte SJ, Kumar A (2010). Alternaria blight: A chronic disease in rapeseed-mustard. J. Oilseed Brassica.

[52] Levin DA (1973). The role of trichomes in plant defense. Q. Rev. Biol..

[53] Hanley ME, Lamont BB, Fairbanks MM, Rafferty CM (2007). Plant structural traits and their role in anti-herbivore defence. Perspect. Plant Ecol..

[54] Gessner MO, Schmitt AL (1996). Use of solid-phase extraction to determine ergosterol concentrations in plant tissue colonized by fungi. Appl. Environ. Microbiol..

[55] Beni A, Soki E, Lajtha K, Fekete I (2014). An optimized HPLC method for soil fungal biomass determination and its application to a detritus manipulation study. J. Microbiol. Meth..

[56] Hadacek F, Greger H (2000). Testing of antifungal natural products: Methodologies, comparability of results and assay choice. Phytochem. Anal..

[57] Schrieber K, Schweiger R, Kröner L, Müller C (2019). Inbreeding diminishes herbivore-induced metabolic responses in native and invasive plant populations. J. Ecol..

[58] R Core Team. *R: A language and environment for statistical computing.* R foundation for Statistical Computing, Vienna, Austria. https://www.R-project.org/ (2018).

[59] Bates D, Mächler M, Bolker BM, Walker SC (2015). Fitting linear mixed-effects models using lme4. J. Stat. Softw..

[60] Arnholt, A. T. & Evans, B. *BSDA: Basic statistics and data analysis*. R package version 1.2.0. https://CRAN.R-project.org/package=BSDA (2017).

[61] Fox, J. & Weisberg, S. *An {R} companion to applied regression*. 2 edn. (Sage, 2011) https://socialsciences.mcmaster.ca/jfox/Books/Companion/ (2018).

