# Supplementary material for: Interactions of Bunias orientalis plant chemotypes and fungal pathogens with different host specificity in vivo and in vitro
Source: Sci Rep. 2020 Jul 1;10:10750. doi: 10.1038/s41598-020-67600-7 (PMC7330031; doi:10.1038/s41598-020-67600-7)
Supplement: Supplementary file 1 — Supplementary information [file 41598_2020_67600_MOESM1_ESM.pdf]

Interactions of *Bunias orientalis* plant chemotypes and fungal pathogens with different host specificity *in vivo* and *in vitro*

Lisa Johanna Tewes[1], Caroline Müller[1]

[1]Department of Chemical Ecology, Bielefeld University, Universitätsstr. 25, 33615 Bielefeld, Germany

Supplementary Information Methods S1, S2, S3, Figs S1, S2 and Table S1

Methods S1: Details on experimental plant rearing

Seeds were collected from the original sites by collaborating scientists and 11-16 plants per population were cultivated in a common garden in Germany (Bielefeld, 52°2.022'N, 8°29.718'E), whereby cross-pollination between populations was prevented. A F1 generation of seeds was collected in the second year, in which the perennial plants flowered the first time. After vernalisation at 4 °C for several months, seeds were germinated on moist filter paper. When the cotyledons had fully developed, seedlings were transferred to 0.6-L pots with a 1:1 mixture of autoclaved seedling soil (Archut Fruhstorfer Erde Typ LAT-Terra Standard Pickiererde; Hawita, Vechta, Germany) and potting soil (C 710 with Cocopor, Stender, Schermbeck, Germany). Per chemotype, a total of 30 seedlings were used for the experiments 10-14 weeks after transfer. Plants were maintained in a climate cabinet (22 °C, 75% relative humidity, 16:8 h light:dark cycle), watered if necessary and fertilised once per week with 10 mL Wuxal Super (1 mL L^{-1}, Aglukon Spezialdünger, Düsseldorf, Germany) for five weeks prior to the experiment.

Methods S2: Details on ergosterol analysis

Detailed protocol for ergosterol extraction

For ergosterol extraction, the lyophilised infested leaf samples and negative controls were pulverised in a ball mill (MM302, Retsch, Haan, Germany). The dry mass of the leaf samples was on average 50 mg (range 37 to 81 mg). To each sample 1 mL methanol (LC-MS grade, Promochem, LGC Standards, Wesel, Germany) was added and samples were extracted for 60 min in an ultrasonic bath, which was cooled by adding crushed ice to the water. The samples

were centrifuged, the supernatant was collected, and the extraction procedure was repeated twice with 500 µL methanol per extraction step. The combined supernatants were dried in a rotary evaporator (RVC 2-18, Christ, Osterode, Germany) at 35 °C, and stored at 4 °C over night. The dried extracts were re-suspended in 250 µL methanol for 30 min in the cooled ultrasonic bath, centrifuged, filtered (polytertafluoroethylene membrane 0.2 µL, 4 mm diameter; Phenomenex, Aschaffenburg, Germany), and used for chemical ergosterol quantification.

Details on HPLC-DAD analysis of ergosterol

Ergosterol was analysed using high performance liquid chromatography equipped with a ZORBAX Eclipse Plus C18 column (250 × 4.6 mm, 5 µm particle size, Agilent) and coupled with a diode array detector (1260 and 1290 Series, Agilent, Santa Clara, CA, USA). For separation of the plant extract 40 µL per sample was injected at a column temperature of 45 °C. A gradient from millipore water (solvent A) to a 1:1 [v/v] mixture (solvent B) of methanol (LC-MS grade, Chemsolute, Th. Grayer, Renningen, Germany) and acetonitrile (LC-MS grade, VWR, Fontenay-sous-Bois, France) was applied at a flow rate of 1 mL min^{-1}, increasing solvent B from 5% B to 100% B within 2 min, holding 100% B for 20 min, followed by a cleaning and equilibration cycle. The UV-absorption of ergosterol was quantified at 282 nm and related to a linear seven-point calibration curve (100, 50, 10, 5, 1, 0.5 and 0.1 µg mL^{-1}) from a commercial ergosterol reference standard (Acros organics, Geel, Belgium) solved in 100% methanol. The detection limit was below 0.004 µg in 40 µL injection volume in the reference standard, and the yield of ergosterol was at least 1 µg per mg pure mycelium mixed with leaf powder, resulting in a theoretical recovery of at least 0.05 mg fungal biomass in the matrix of leaf material.

Methods S3: Details on UHPLC-TOF analysis of leaf material

Leaf extract samples were analysed using ultra high performance liquid chromatography (Dionex UltiMate 3000, Thermo Fisher, San José, CA, USA) coupled to a quadrupole time of flight mass spectrometer (compact, Bruker Daltonics, Bremen, Germany). Sample preparation and chemical analysis were performed according to Schrieber et al. (2019), but with deviations in the chemical analysis method and the respective treatment of the raw data from chromatograms. The ionisation was done in negative electrospray ionisation mode with a capillary voltage of 3,000 V, and with different settings for the collision cell (pre-pulse

storage: 6 µs, transfer time 75 µs). Peak picking from the chromatograms within the 'find molecular features' function allowed a signal-to-noise threshold of 1. Bucket generation in the dataset from negative electrospray ionisation mode allowed the ion types $[M-H]^-$, $[M-H_2O-H]^-$, $[M+Cl]^-$, $[M+HCOOH-H]^-$, $[M+CH_3COOH-H]^-$, $[2M-H]^-$, $[2M+HCOOH-H]^-$, $[2M+CH_3COOH-H]^-$ and $[3M-H]^-$, and bucketing was done allowing m/z deviations of 6 mDa with no pre-filter for bucket counts. Glucosinolates were identified from the dataset by their mass spectra compared to internal databases and quantified by relating the peak area of the maximum isotope m/z to response factors for different side chain types (aliphatic glucosinolates: 0.86, *p*-hydroxybenzyl glucosinolate: 1, benzyl glucosinolate: 0.62, indole glucosinolates: 1), to the internal standard hydrocortisone (response factor 0.86), and to the sample dry weight according to Tewes et al. (2018).

References

Schrieber, K., Schweiger, R., Kröner, L. & Müller, C. Inbreeding diminishes herbivore-induced metabolic responses in native and invasive plant populations. *J. Ecol.* **107**, 923-936, doi:10.1111/1365-2745.13068 (2019).

Tewes, L. J., Michling, F., Koch, M. A. & Müller, C. Intracontinental plant invader shows matching genetic and chemical profiles and might benefit from high defence variation within populations. *J. Ecol.* **106**, 714-726, doi:10.1111/1365-2745.12869 (2018).

Figure S1: Chromatogram of ergosterol analysis

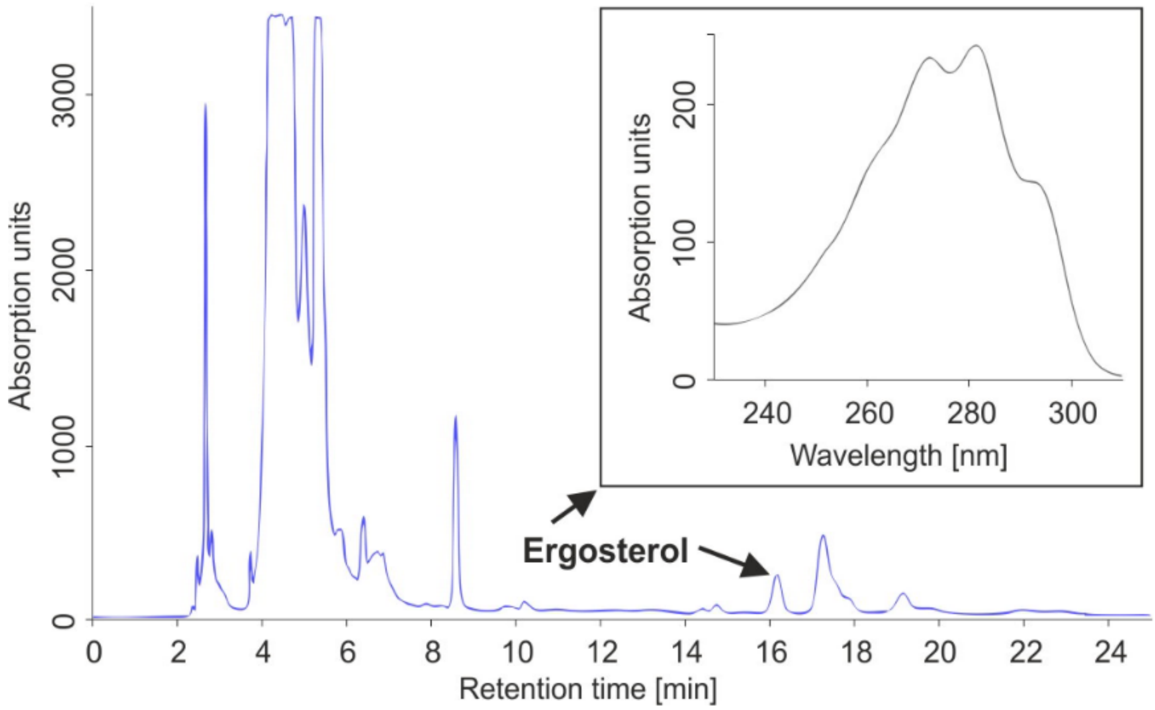

Fig. S1 Typical chromatogram of UV absorbance at 282 nm in extracts of *Bunias orientalis* leaves infested with fungal pathogens, measured with high pressure liquid chromatography coupled with a diode array detector. The peak of ergosterol as biomarker for fungal biomass is indicated at ~16.1 min retention time. The typical UV spectrum of ergosterol used for identification is shown within the box.

Figure S2: Chromatograms of fractioned leaf extract

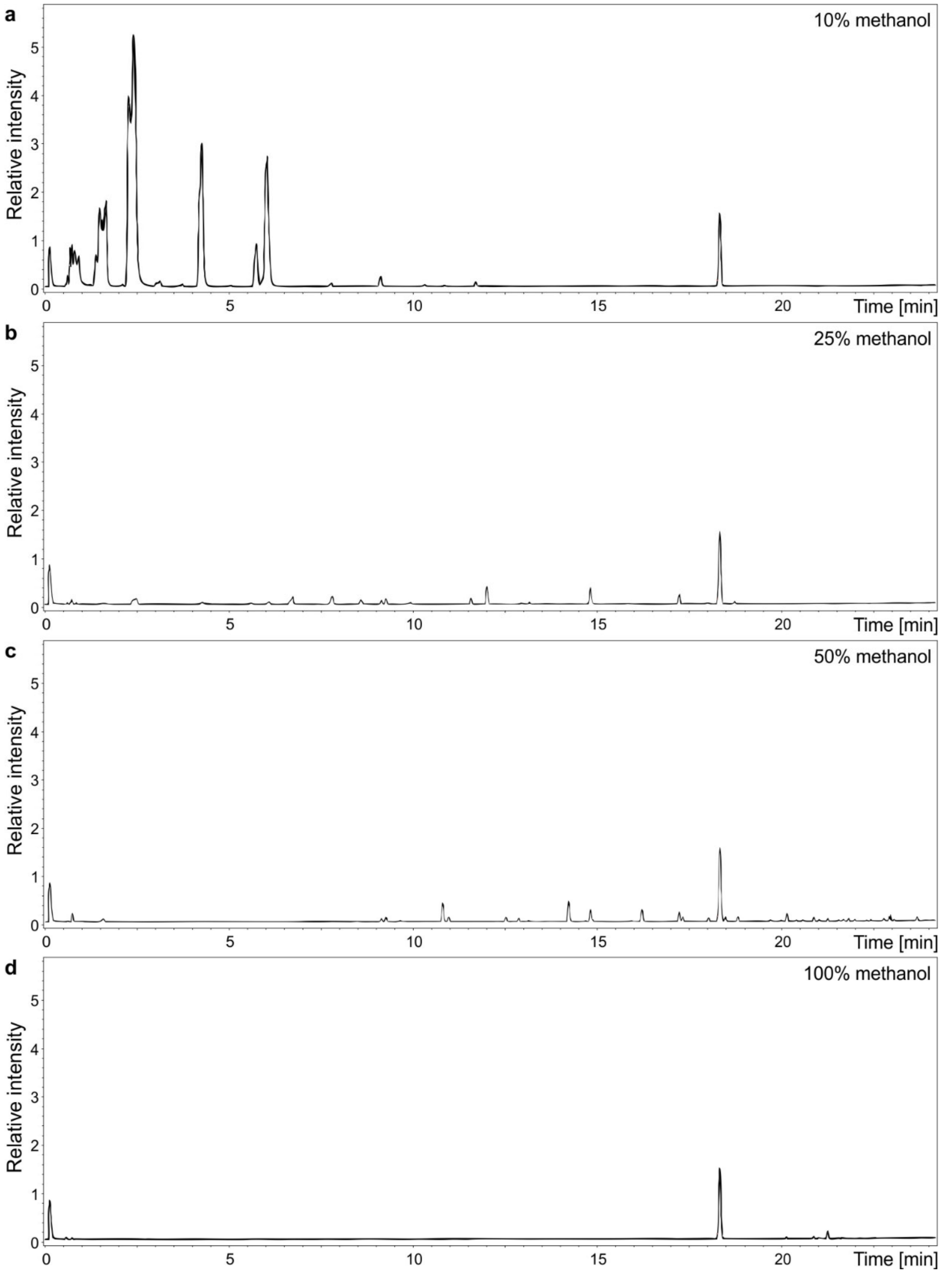

Fig. S2 Chromatograms of fractions of a methanol extract of leaves from *Bunias orientalis* plants. The fractions were gained by solid phase extraction and analysed using ultra high performance liquid chromatography coupled with time-of-flight mass spectrometry. Solid phase extraction columns were successively washed with methanol:water mixtures of **(a)** 10% methanol, **(b)** 25% methanol, **(c)** 50% methanol and **(d)** 100% methanol. The internal standard hydrocortisone added to each sample elutes at ~18.4 min.

Table S1 Statistics on relative mycelium growth change of the fungal pathogens *Alternaria brassicae* and *Botrytis cinerea* in liquid nutrient broth amended with extracts of leaf material of *Bunias orientalis* in concentrations of 100 and 200 ppm. Extracts were made from leaf material from plants of different chemotypes ('Turkish', 'German') in which leaves of 30 plants per chemotype were pooled. Samples in which leaf material from both chemotypes was combined (n = 60 plants) was fractionated using solid phase extraction with different methanol:water mixtures to achieve four fractions. Fungal biomass was measured as optical density three days after subjecting conidia to amended nutrient broth. The percentage of growth change in relation to means of control samples without plant extracts was calculated and growth change values were tested for being significantly from zero (i.e. the control mean values) in one-sided sign tests. Significant P values are highlighted in bold.

		Alternaria brassicae			*Botrytis cinerea*		
Chemotype	ppm	n	S	P	n	S	P
Turkish	100	10	5	1	9	9	**0.004**
	200	9	1	**0.039**	10	10	**0.002**
German	100	8	3	0.726	10	10	**0.002**
	200	9	9	**0.004**	10	10	**0.002**
Fraction	ppm	n	S	P	n	S	P
10% Methanol	100	10	4	0.754	7	1	0.125
	200	10	9	**0.022**	9	0	**0.004**
25% Methanol	100	10	5	1	10	6	0.754
	200	10	9	**0.022**	9	7	0.180
50% Methanol	100	10	5	1	9	4	1
	200	10	8	0.109	10	4	0.754
100% Methanol	100	10	4	0.754	10	8	0.109
	200	10	0	**0.002**	9	7	0.180
